# Molecular detection and characterization of the *Mycobacterium tuberculosis* complex subspecies responsible for bovine tuberculosis in Punjab, Pakistan

**DOI:** 10.1128/spectrum.02692-23

**Published:** 2024-01-16

**Authors:** Rubab Maqsood, Shannon C. Duffy, Hamad Bin Rashid, Shakera Sadiq Gill, Chanda Jabeen, Nimra Arshad, Gulshan Umbreen, Marcel A. Behr, Vivek Kapur, Mamoona Chaudhry

**Affiliations:** 1 Department of Epidemiology and Public Health, University of Veterinary and Animal Sciences, Lahore, Pakistan; 2 Institute of Continuing Education and Extension, University of Veterinary and Animal Sciences, Ravi Campus, Pattoki, Pakistan; 3 Department of Microbiology and Immunology, McGill University, Montreal, Quebec, Canada; 4 The Infectious Diseases and Immunity in Global Health Program, Research Institute of the McGill University Health Centre, Montreal, Quebec, Canada; 5 Department of Veterinary Surgery and Pet Sciences, University of Veterinary and Animal Sciences, Lahore, Pakistan; 6 Department of Medicine, McGill University, Montreal, Quebec, Canada; 7 Department of Animal Science, The Pennsylvania State University, University Park, Pennsylvania, USA; 8 The Huck Institutes of the Life Sciences, The Pennsylvania State University, University Park, Pennsylvania, USA; NHLS Tygerberg/Stellenbosch University, South Africa

**Keywords:** bovine TB, *M. orygis*, *M. tuberculosis*, slaughterhouse, molecular identification, whole-genome sequencing

## Abstract

Bovine tuberculosis (bTB), traditionally associated with *Mycobacterium bovis*, presents significant public health and economic challenges worldwide. This study investigated the causative agents of bTB in slaughtered cattle and buffalo in Lahore, Pakistan. Of the 3,581 animals screened, 34 were identified with gross TB-like lesions. The lesions were processed for culture, PCR, and Sanger sequencing to identify the causative agents of the disease. The results identified 10 *Mycobacterium orygis* and 8 *Mycobacterium tuberculosis sensu stricto* isolates. Whole-genome sequencing was performed on two *M*. *orygis* isolates, and the sequences were phylogenetically compared to 93 publicly available *M. orygis* sequences. The results also demonstrated that the JB21 and JB22 primers, which have been previously commonly applied to detect *M. bovis* in Pakistan, are unable to distinguish between *M. tuberculosis* complex subspecies. The identification of *M. orygis* and *M. tuberculosis* as causative agents of bTB in this slaughterhouse in Punjab may have important implications in identifying cases of zoonotic TB in humans and applying appropriate molecular tools to identify the prevalence of the disease. The data from this study align with recent findings suggesting *M. orygis* is the predominant cause of bTB in South Asia.

**IMPORTANCE:**

The study findings hold significant relevance to the Journal of Clinical Microbiology, as they directly impact the field. The first-time identification of *Mycobacterium orygis* and *Mycobacterium tuberculosis* as the predominant causative agents of bovine tuberculosis in Lahore, Pakistan underscores the urgent need for enhanced diagnostic methods. The study emphasizes the importance of improved assays for the accurate detection and differentiation of Mycobacterium subspecies. Additionally, the research addresses zoonotic risk assessment and public health implications, advocating for a multidisciplinary approach that integrates clinical microbiology with veterinary and human health sectors. These insights contribute to clinical microbiology knowledge, shaping effective strategies for disease prevention, surveillance, and control. The study’s potential to advance the field makes it well suited for publication in the Microbiology Spectrum journal.

## INTRODUCTION

Bovine tuberculosis (bTB) is a chronic disease that is estimated to affect 7% of the world’s cattle population (1). In low- and middle-income countries, bTB continues to be a serious concern for the health of cattle and other livestock (2) and for public health (3). Countries with endemic bTB face substantial economic losses due to reduced productivity of infected animals (4), condemnation of meat or carcasses at slaughterhouses (5), and constraints on animal trade (6). *Mycobacterium bovis* is the most commonly reported cause of bTB (7–9) and belongs to a related group of mycobacteria known to cause tuberculosis called the *Mycobacterium tuberculosis* complex (MTBC) (10). *Mycobacterium orygis,* a lesser-known MTBC subspecies, has also been reported as a cause of tuberculosis in cattle, particularly within South Asia (11–13). To detect bTB postmortem, histopathological techniques can be used to identify TB in granulomatous or fibrotic tissue lesions of animal tissue, but these are limited in their sensitivity and specificity (14). Alternatively, the detection of bTB using culture is a laborious process that takes weeks. Molecular assays have been proven to be a sensitive, reliable, and rapid method for the identification of bTB (15, 16). They also offer the additional benefit of being able to differentiate between MTBC subspecies to determine the cause of the disease. Slaughterhouses may serve as sentinel sites for monitoring various diseases of animals and can also provide insight into the prevalence of bTB (17). In Pakistan, studies on the prevalence of bTB have varied by region, sample size, sampling strategy, and analysis, with estimates ranging from 14.00% (18) to 2.71% (19). Throughout Pakistan, *M. bovis* has previously been reported as the sole cause of zoonotic (20) and bovine TB (21). The majority of investigations in Pakistan used PCR with the JB21 and JB22 primers to detect zoonotic or bovine TB in human and animal samples (22–24). These primers were previously reported to amplify an *M. bovis*-specific 500-bp sequence (25).

In this study, we aimed to perform molecular assays to identify MTBC subspecies recovered from TB-like lesions in cattle and buffalo at a slaughterhouse in Lahore, Pakistan. We hypothesized that bTB would be present and would be caused by *M. bovis*. We applied molecular assays, whole-genome sequencing (WGS), and phylogenetic analysis to characterize the MTBC subspecies collected in our study. To our knowledge, this is the first report of whole genome sequences from cases of bTB in Pakistan. Identification of the etiological agents of bTB in the region may be important for understanding potential zoonotic transmission, estimating the prevalence of disease, and developing appropriate diagnostics for its detection. The results of this study may, therefore, better inform control policies for zoonotic and bovine TB in the region.

## MATERIALS AND METHODS

### Study area

In the district of Lahore, which is the capital of Punjab, there are three public and five private sector red meat slaughterhouses. The single largest public-sector slaughterhouse in the city of Lahore (31°32′59″N 74°20′37″E) was selected for the present study to accommodate logistics and accessibility issues. The slaughterhouse is adjacent to one of the biggest live animal markets (locally called maweshi mandi) in Punjab where animal traders (locally called Beopari) routinely bring animals for sale from different neighboring cities of Lahore. The majority of beef contractors/traders from the slaughterhouse purchase animals from this adjacent live animal market. This slaughterhouse is the major catchment area for the animals brought for slaughter in Lahore.

### Study population and design

A cross-sectional study was conducted for 5 months (November 2021–March 2022) at the selected slaughterhouse. The study population comprised 3,581 animals (441 cattle and 3,140 buffalo) slaughtered at the site within the study period. Animals slaughtered in slaughterhouses belonging to the corporate sector were excluded from the target population as they prefer to slaughter young male animals from feedlot fattening farms. In the lairage, animals were subjected to antemortem examination, and information about the species, age, and gender of the animals was recorded. All animals were declared fit for slaughter at antemortem. Beef contractors/traders from the slaughterhouse were also interviewed individually about the point of purchase of animals to assess the spatial distribution of slaughtered animals. Geographical information was only available for 772 animals.

### Sample collection and processing

Initial postmortem inspection of carcasses resulted in the collection of specimens, mostly lymph nodes (pharyngeal, retropharyngeal, and mediastinal), lungs, and liver, presenting any kind of superficial visible lesions or abscesses. All collected samples were then examined thoroughly for the presence of granulomatous gross visible lesions attributable to tuberculosis and declared clinically suggestive if present (9, 26). Tissues with TB-like lesions were sealed in sterile pre-labeled plastic containers and were transported at refrigerated conditions to the laboratory at the Department of Epidemiology and Public Health, University of Veterinary and Animal Sciences, Lahore where these samples were stored at 4°C (9).

In the laboratory, TB-like lesions from necropsy samples from each carcass were triturated and divided into two aliquots. One aliquot was transported to the Provincial TB Reference Laboratory, Lahore for a Mycobacterial Growth Indicator Tube (MGIT) culture test using the BACTEC MGIT 960 instrument. One sample (bR3) was cultured on Lowenstein-Jensen (LJ) media when MGIT stock was low. This reference laboratory primarily handles human tuberculosis samples under the Primary and Secondary Healthcare Department, Ministry of Health Punjab, Pakistan. If the sample was culture-positive, DNA was extracted by boiling. The second aliquot was suspended in lysis buffer and placed for overnight incubation in a dry hot bath. The DNA extraction was performed using phenol-chloroform-isoamyl alcohol (PCI) and the ethanol precipitation method (Supplementary Methods), and the eluted DNA was stored at −20°C (27).

### PCR analysis

A PCR that has been previously used to identify *M. bovis* was first performed at the University of Veterinary and Animal Sciences, Lahore. This PCR used a set of primers (JB21 and JB22) to generate a 500-bp fragment reported to be specific to *M. bovis* (25) (Supplementary Methods; Table S1). An annealing temperature of 55°C was used as opposed to the 68°C annealing temperature from reference (25). During PCR optimization, no amplicon was generated using 68°C, so a gradient PCR was performed using a 0.5°C difference interval. This found that the product could be generated using 55°C.

DNA from all samples was also sent to the Research Institute at the McGill University Health Centre in Montreal for additional analysis of the MTBC subspecies collected. Samples were analyzed using two previously described PCR assays, which differentiate between *M. tuberculosis sensu stricto, M. bovis/M. bovis* BCG, and *M. orygis* (28). The first assay is a three-primer PCR, which detects the presence or absence of region of difference (RD) 9. A ~200 bp fragment is amplified if RD9 is present and a ~400 bp fragment is amplified if it is absent. *M. tuberculosis sensu stricto* has RD9 and *M. bovis/M. bovis* BCG and *M. orygis* do not. The second assay is a six-primer PCR, which detects differences in the deletion size of RD12 (Supplementary Methods; Table S1). RD12 is found in *M. tuberculosis sensu stricto* and absent in *M. bovis/M.bovis* BCG and *M. orygis* with the deletion size being larger in *M. orygis*. Using this PCR, a ~400 bp fragment is amplified if RD12 is present, a ~600 bp fragment is amplified if the *M. bovis*/*M. bovis* BCG size deletion is present, and a ~250 bp fragment is amplified if the *M. orygis* size deletion is present. Samples that did not produce an amplicon on either the RD9 or RD12 assay were subject to PCR of the 16S or hsp65 regions followed by Sanger sequencing to confirm their identity (Table S1) (29, 30). The samples were initially tested using primers targeting the hsp65 sequence. If the sample was negative, the PCR was repeated using primers to detect the 16S sequence.

### Whole-genome sequencing

Samples prepared by the PCI and ethanol precipitation method and which had been identified as an MTBC subspecies by PCR were submitted for confirmation by WGS. These samples were chosen due to the preparation method of the DNA samples. Ideally, DNA would have been prepared from the positive cultures using a method compatible for WGS, but this was not available. The Illumina S4 reagent kit was used to prepare paired-end sequencing libraries, and shotgun sequencing was performed on the Illumina NovaSeq 6000. Sequences were analyzed using the validated SNP (vSNP) pipeline (available at https://github.com/USDA-VS/vSNP) developed by the United States Department of Agriculture-Veterinary Services to align sequences to *M. tuberculosis* H37Rv (NC_000962.3) and assign an MTBC subspecies identity as has been previously described (28). Integrative Genomics Viewer was used to visualize sequences (31). Sequences were deposited in the National Center for Biotechnology Information (NCBI) Sequence Read Archive (SRA) under the BioProject accession number PRJNA958208.

### Phylogenetic tree assembly

Newly collected sequences were phylogenetically compared with other publicly available *M. orygis* sequences available on the SRA database (Table S2). A phylogenetic tree file was generated by vSNP and rooted to *M. tuberculosis* H37Rv (NC_000962.3). The tree was visualized and annotated using the interactive tree of life (version 6) (32).

### Spatial distribution analysis

QGIS version 3.28.2 Bonn (available at http://qgis.org/) was used to generate the map for the spatial distribution of slaughtered animals.

## RESULTS

### Study population

**Fig 1:**
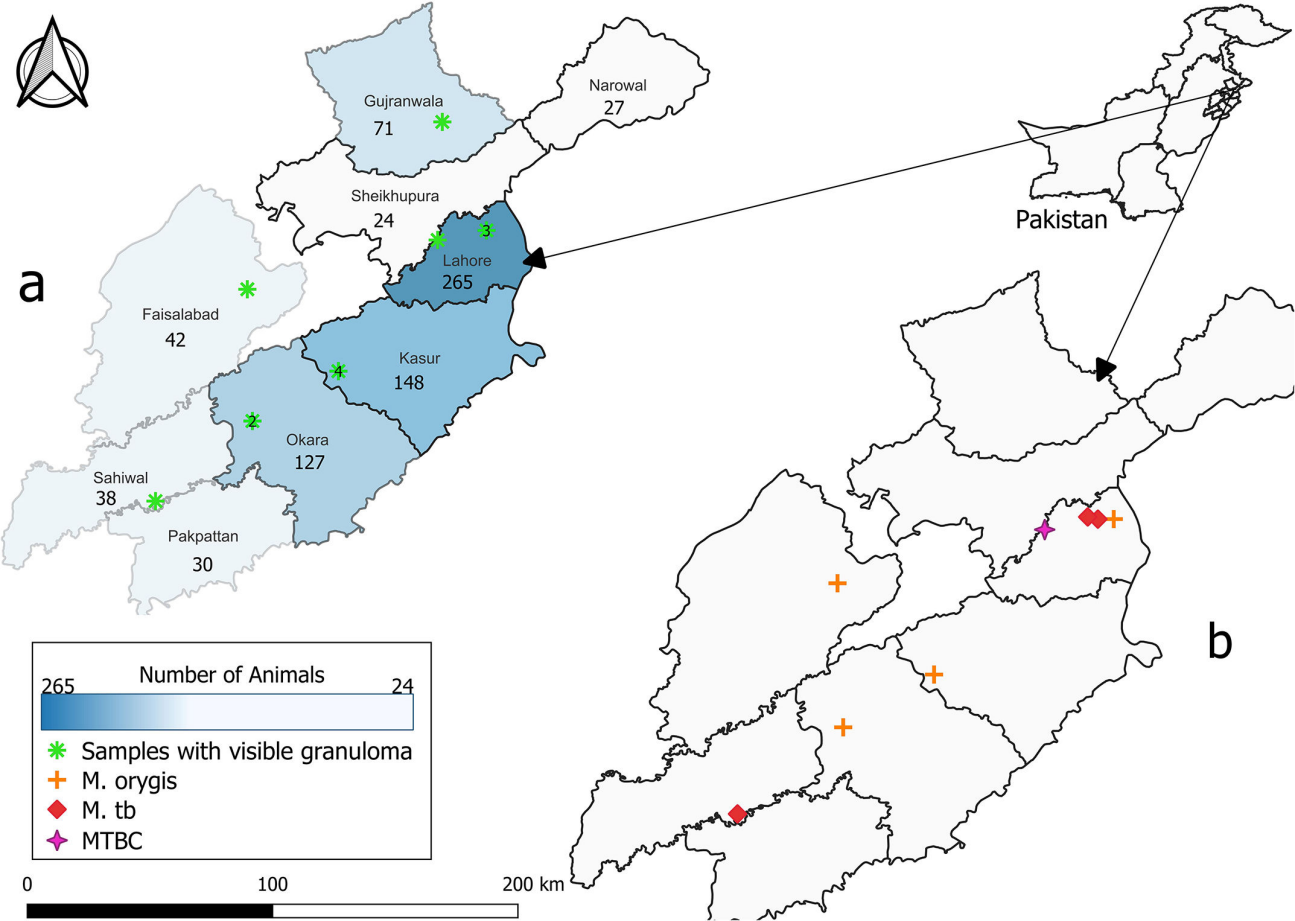
Spatial distribution of 772 screened bovines in nine districts of Punjab Province, Pakistan. Geographic information was only available for 772 of the 3,581 total animals in the study. The color of each district in map “a” shows the number of animals screened from that location. The specific number collected from each district is listed below the district name. Samples from the Lahore peripheries are listed within the Lahore district. Map “a” also identifies the locations where samples with visible granulomas came from (*n* = 13). If more than one sample came from that location, the number is listed on top of the green symbol. Map “b” shows the distribution of eight MTBC subspecies with location information available, which were identified in carcasses with visible granulomas: four were *M. orygis,* three were *M. tuberculosis*, and one MTBC.

A total of 3,581 large animals were brought to the selected slaughterhouse. The location of purchase information was only available from 59 beef contractors for 772 (21.56%) animals. Of those, animals were brought from nine different districts: Lahore peripheries (*n* = 265), Narowal (*n* = 27), Shekhupura (*n* = 24), Gujranwala (*n* = 71), Kasur (*n* = 148), Okara (*n* = 127), Pakpattan (*n* = 30), Sahiwal (*n* = 38), and Faisalabad (*n* = 42) (Fig. 1). Of the remaining 2,809 animals, 1,471 came from a live animal market, which were pooled from different districts of Punjab, and no information was available for the other 1,338. Of the total 3,581 animals, 3,140 (87.68%) samples were from buffaloes and 441 (12.31%) were from cattle carcasses. The majority (*n* = 3,212) of the large animals screened were females (89.69%).

### Examination of animals for gross TB-like lesions

**Fig 2:**
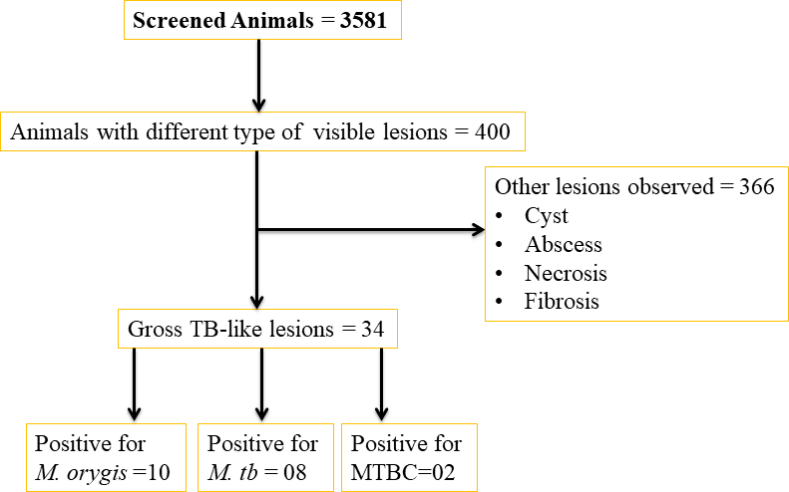
Sample selection and characteristics.

Throughout the study period, a total of 400 carcasses were selected based on the presence of any type of lesion (granulomas, necrosis, abscess, fibrosis, and cysts) on their tissues (Fig. 2). Upon thorough examination of those 400 carcasses for lesions clinically suggestive of tuberculosis based on the presence of gross TB-like lesions or tubercles, only 34 samples were identified (Fig. 2; Table 1). Of these samples, 30 were from buffalo (28 females and 2 males) and four were from cattle carcasses (all female). TB-like lesions were observed on the lungs (*n* = 28), lymph nodes (*n* = 13), and liver (*n* = 3).

**TABLE 1:** Characteristics of bTB samples collected from November 2021 to March 2022 from the slaughterhouse in Lahore, Pakistan

Sample	Species	Location^a^	Age (years)	Sex^b^	Tissue type^c^	Culture^d^
bR1	Buffalo	NA	2	M	Lungs	P
bR2	Buffalo	Okara	5	F	Lungs + LNs	P
bR3	Buffalo	NA	8	F	Liver	P*
bR4	Buffalo	NA	7	F	Lungs	P
bR5	Cattle	Kasur	10	F	Lungs	P
bR6	Buffalo	Lahore peripheries	4	F	Lungs	P
bR7	Buffalo	Kasur	9	F	Lungs	P
bR8	Buffalo	Lahore peripheries	10.5	F	Lungs + LNs	P
bR9	Buffalo	Sahiwal	11	F	Lungs	P
bR10	Buffalo	NA	7	F	Liver	P
bR11	Buffalo	NA	9	F	Lungs	P
bR12	Buffalo	Faisalabad	4.5	F	Lungs	P
DR1	Buffalo	NA	10	F	Lungs + LNs	NA
DR2	Cattle	NA	9	F	Lungs + LNs	NA
DR3	Buffalo	NA	11	F	Lungs	N
DR4	Buffalo	NA	6	F	Lungs + LNs	N
DR5	Buffalo	NA	10	F	Lungs	N
DR6	Buffalo	NA	12	F	Lungs	N
DR7	Buffalo	NA	4	F	Lungs + LNs	NA
DR8	Buffalo	Gujranwala	4.5	F	Lungs	NA
DR9	Buffalo	Lahore peripheries	6.5	F	Lungs	NA
DR10	Buffalo	NA	7	F	LNs	NA
DR11	Cattle	Kasur	8	F	Lungs	NA
DR12	Buffalo	Okara	9	F	Lungs	NA
DR13	Buffalo	NA	7.5	F	Lungs + LNs	NA
DR14	Buffalo	NA	8	F	Lungs	NA
DR15	Buffalo	NA	7	F	Lungs + LNs	NA
DR16	Buffalo	NA	9.5	F	Lungs	NA
DR17	Buffalo	Kasur	6	F	LNs	NA
DR18	Cattle	NA	8	F	Lungs	NA
DR19	Buffalo	Lahore peripheries	10	F	Lungs + LNs	NA
DR20	Buffalo	NA	1.5	M	Liver + LNs	NA
DR21	Buffalo	NA	NA	F	Lungs	NA
DR22	Buffalo	NA	8.5	F	LNs	NA

^a^ NA, location not available.

^b^ Gender of sampled animals: F, female and M, male.

^c^ Types of tissues collected: LNs, lymph nodes.

^d^ Culture results: P, positive by MGIT; P*, positive by LJ; N, negative; and NA, culture results not available.

### Culture of samples

Culture test was performed on samples from 16 of the 34 animals with TB-like lesions that were submitted to the Provincial TB Reference Laboratory. Due to a power outage, the lab was unable to perform the culture test for the remaining 18 samples. Of the 16 cultured samples, 12 of these were positive on MGIT or LJ (Table 1).

### PCR results

In the initial conventional PCR screening conducted in Lahore using JB21 and JB22 primers, five were positive (Table 2; Fig. S1). Further analysis of the samples using the RD9 and RD12 assays indicated that 18 of the 34 samples were positive for MTBC subspecies: 8 of which were identified as *M. tuberculosis sensu stricto* and 10 of which were identified as *M. orygis*. No samples were identified as *M. bovis*. The 16 remaining samples produced no band on either the RD9 or RD12 assays. These samples were subjected to PCR of the 16S or hsp65 sequences followed by Sanger sequencing to confirm their identity: eight produced no band on either PCR indicating a lack of bacterial DNA present, six were identified by 16S PCR and Sanger sequencing as non-mycobacterial species, and two were identified as MTBC following hsp65 PCR and Sanger sequencing. The RD9 and RD12 assays were re-attempted twice on the two samples identified as MTBC by hsp65 PCR but returned negative results each time. These samples were, therefore, classified as MTBC (Table 2). The complete workflow description of how samples were selected and screened is given in Fig. S2.

**TABLE 2:** PCR identification of bTB samples collected from November 2021 to March 2022 from the slaughterhouse in Lahore, Pakistan

Sample^a^	DNA preparation method	JB21/22^b^ (bp)	RD9 PCR^c^ (bp)	RD12 PCR^d^ (bp)	Sanger sequencing^e^	Identification
bR1	Boiling	500	200	400		*M. tuberculosis*
bR2	Boiling	500	400	250		*M. orygis*
bR3	Boiling	500	200	400		*M. tuberculosis*
bR4	Boiling	N	400	250		*M. orygis*
bR5	Boiling	N	N	N	*Cohnella* species	*Cohnella* species
bR6	Boiling	N	200	400		*M. tuberculosis*
bR7	Boiling	N	N	N	*Streptococcus* species	*Streptococcus* species
bR8	Boiling	500	200	400		*M. tuberculosis*
bR9	Boiling	500	200	400		*M. tuberculosis*
bR10	Boiling	N	200	400		*M. tuberculosis*
bR11	Boiling	N	400	250		*M. orygis*
bR12	Boiling	N	400	250		*M. orygis*
DR1	PCI/EtOH	N	200	400		*M. tuberculosis*
DR2	PCI/EtOH	N	400	250		*M. orygis*
DR3	PCI/EtOH	N	400	250		*M. orygis*
DR4	PCI/EtOH	N	N	N	N	Unknown
DR5	PCI/EtOH	N	N	N	N	Unknown
DR6	PCI/EtOH	N	N	N	*Fusobacterium* species	*Fusobacterium* species
DR7	PCI/EtOH	N	400	250		*M. orygis*
DR8	PCI/EtOH	N	N	N	N	Unknown
DR9	PCI/EtOH	N	400	250		*M. orygis*
DR10	PCI/EtOH	N	N	N	*Trueperella pyogenes*	*Trueperella pyogenes*
DR11	PCI/EtOH	N	N	N	*Bacteriodes heparinolyticus*	*Bacteriodes heparinolyticus*
DR12	PCI/EtOH	N	N	N	N	Unknown
DR13	PCI/EtOH	N	N	N	N	Unknown
DR14	PCI/EtOH	N	N	N	N	Unknown
DR15	PCI/EtOH	N	N	N	N	Unknown
DR16	PCI/EtOH	N	N	N	*Moxarella bovoculi*	*Moxarella bovoculi*
DR17	PCI/EtOH	N	400	250		*M. orygis*
DR18	PCI/EtOH	N	N	N	N	Unknown
DR19	PCI/EtOH	N	N	N	MTBC	MTBC
DR20	PCI/EtOH	N	400	250		*M. orygis*
DR21	PCI/EtOH	N	200	400		*M. tuberculosis*
DR22	PCI/EtOH	N	N	N	MTBC	MTBC

^a^ bR, culture positive and DNA preparation was done using the boiling method and DR, DNA extracted directly from tissue lesions using PCI and ethanol precipitation method.

^b^ N, negative results for PCR with JB21 and JB22 primers.

^c^ N, negative for RD9 PCR.

^d^ N, negative for RD12 PCR.

^e^ N, negative for Sanger sequencing.

### Validation of JB21 and JB22 primers

**Fig 3:**
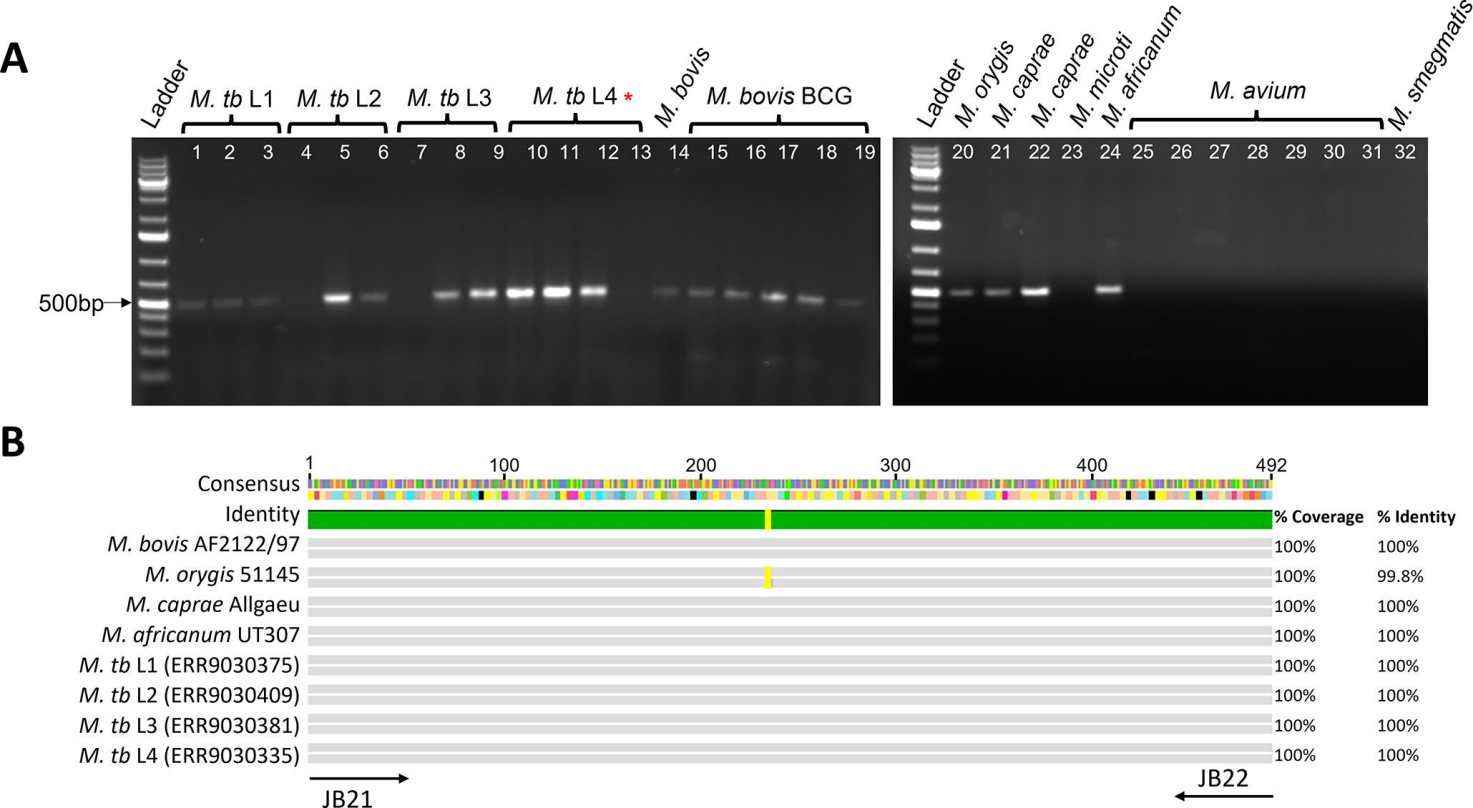
Amplification in different MTBC subspecies using the JB21 and JB22 primers. (**A)** A PCR was performed using DNA from a variety of MTBC subspecies to determine the specificity of the JB21 and JB22 primers. A 1 Kb plus DNA ladder (Invitrogen) was loaded on the gel for screening. The expected product was 500 bp. Lanes 1–3: *M. tuberculosis* lineage 1. Lanes 4–6: *M. tuberculosis* lineage 2. Lanes 7–9: *M. tuberculosis* lineage 3. Lanes 10–12: *M. tuberculosis* lineage 4. Lane 13: *M. tuberculosis* H37Rv (denoted by red asterisk). Lane 14: *M. bovis* AF2122/97. Lanes 15–19: BCG Russia, BCG Danish, BCG Sweden, BCG Glaxo, and BCG Prague. Lane 20: *M. orygis* 51145. Lanes 21–22: *M. caprae* BC and *M. caprae* 65749. Lane 23: *M. microti*. Lane 24: *M. africanum* MT18 7400. Lanes 25–31: *M. avium* subsp. *avium* 15769, *M. avium* subsp. *avium* 35718, *M. avium* subsp. *avium* 35713, *M. avium* subsp. *hominissuis* 104, *M. avium* subsp. *paratuberculosis* K10, *M. avium* subsp. *paratuberculosis* Holland, *M. avium* subsp. *paratuberculosis* 6758. Lane 32: *M. smegmatis* MC^2^ 155. (**B)** An alignment of the 492-bp sequence amplified by JB21 and JB22 in different MTBC subspecies using Geneious Prime.

Given that no *M. bovis* was identified by the RD9 or RD12 assays, this prompted investigation of the JB21 and JB22 primers, which have been previously reported to amplify an *M. bovis*-specific fragment and have repeatedly been used to identify *M. bovis* in studies within Pakistan (20–24, 33–39). A PCR was performed with the JB primers using DNA from various confirmed mycobacteria including several *M. tuberculosis* lineages (three to four isolates per lineage) including H37Rv, *M. bovis* AF2122/97, five *M*. *bovis* BCG strains, *M. orygis, Mycobacterium caprae, Mycobacterium microti, Mycobacterium africanum,* and eight non-tuberculosis mycobacteria. The expected 500-bp band was produced for the *M. bovis* and *M. bovis* BCG strains. However, amplification was also observed with DNA from several *M. tuberculosis* strains, *M. orygis, M. caprae*, and *M. africanum* (Fig. 3A). Further analysis of the amplicon using the Basic Local Alignment Search Tool (BLAST) from NCBI and visualization via Geneious Prime determined that the JB primers amplify a 492-bp fragment of a helicase gene: *Mb2049c* in *M. bovis* AF2122/97 or *RJtmp002096* in *M. orygis* 51145. Specifically, the primers amplify nucleotides 2,253,648–2,254,140 in *M. bovis* and 2,249,132–2,249,624 in *M. orygis*. This sequence was not found in *M. tuberculosis* H37Rv but was identified in other *M. tuberculosis* isolates (Fig. 3B). This analysis confirmed that the JB primers are not a reliable method for distinguishing *M. bovis* from other MTBC subspecies.

### Whole genome sequencing

A total of 10 DNA samples that were identified as MTBC subspecies by PCR and prepared using the PCI and ethanol precipitation method were submitted for whole-genome sequencing. Of these, three failed library preparation, and five had an inadequate mean depth of coverage and percent genome coverage for analysis. This was likely caused by the low concentration of DNA present in the sample and poor sample purity. The two sequences produced were from samples DR2 and DR20, which had been identified as *M. orygis* by PCR. These sequences have been deposited under the BioProject accession number PRJNA958208. WGS and analysis by the vSNP pipeline confirmed both samples were *M. orygis*. The two sequences were 47 single nucleotide polymorphisms (SNPs) apart, indicating they were likely independently acquired infections (40). Notably, however, the sequence qualities for both samples were still low with a mean depth of coverage of 10× for DR2 and 8× for DR20 and genome coverage of 97.87% for DR2 and 97.73% for DR20. As an added measure, the sequences were checked for the presence of two SNPs known to be specific to *M. orygis: Rv0444c* g698c and a C to T mutation found at nucleotide 2,850,607 (Fig. S3 and S4). Both DR2 and DR20 had these two *M*. *orygis*-specific SNPs. The two *M*. *orygis* sequences were then phylogenetically compared with 93 additional *M. orygis* sequences available on the SRA database, which demonstrated their relatedness to previously published strains (Fig. 3; Table S2) . *M. orygis* sequences included in the tree were isolated from other animals including antelope, cattle, deer, and bison, as well as humans.

Given the combined results of all PCR assays and Sanger sequencing, 20 samples with TB-like lesions were positive for an MTBC subspecies. Of those, 10 were identified as *M. orygis* (50%) and 8 were identified as *M. tuberculosis* (40%). The final two samples (10%) were classified as MTBC because they were identified as MTBC by Sanger sequencing of hsp65, but no bands were produced using the RD9 or RD12 PCRs. The MTBC samples were collected from animals brought to the slaughterhouse from five different districts, indicating that these infections occurred independently (Fig. 1). The proportion of samples positive for MTBC subspecies (*n* = 20) among the clinically suggestive (*n* = 34) was 58.82% (95% CI, 40.93–74.10).

## DISCUSSION

**Fig 4:**
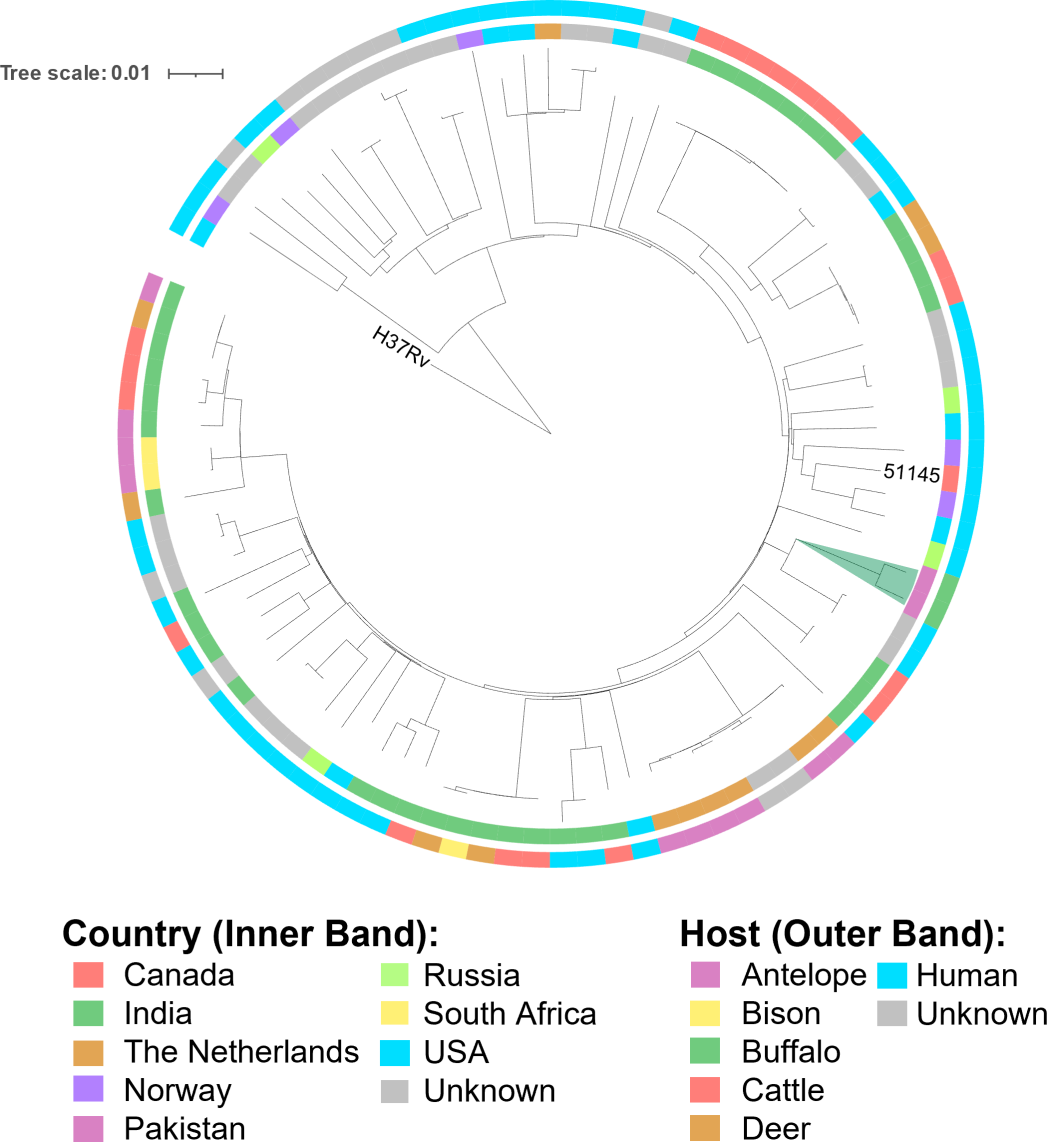
A phylogenetic tree of the two new *M. orygis* sequences from Pakistan and 93 additional publicly available *M. orygis* genomes. The inner band shows the country of origin listed on the NCBI Biosample information for each sequence. The outer band shows the host species. The two sequences from this study are highlighted in green. The reference sequence *M. orygis* 51145 and the root of the tree *M. tuberculosis* H37Rv are labeled.

The World Organization for Animal Health, the World Health Organization, and the Food and Agriculture Organization have long defined *M. bovis* as the causal pathogen of bovine and zoonotic tuberculosis (9). Pakistan is located in South Asia, and bTB is frequently reported from various regions of the country. Previous studies from Pakistan reported *M. bovis* as the sole cause of zoonotic and bTB (21, 41). The majority of these studies concentrated on herd testing and risk factor analysis at the animal and/or herd level (42, 43). This was the first surveillance-based study at a slaughterhouse in Lahore, Pakistan, which aimed to employ PCR assays and sequencing to identify the MTBC subspecies causing bTB in the area. A total of 20 MTBC samples were identified from 34 TB-like lesions: 10 *M*. *orygis,* 8 *M*. *tuberculosis sensu stricto,* and 2 MTBC (Table 2). Two *M. orygis* whole genome sequences were generated from this study and a phylogenetic comparison confirmed their relatedness with other previously deposited *M. orygis* genomes (Fig. 4).

Since our data suggested erroneous detection of *M. bovis* with the JB21 and JB22 primers, we analyzed a panel of confirmed MTBC isolates with these primers. Our results indicated that amplification of the 500-bp product does occur in other MTBC subspecies other than *M. bovis* including *M. tuberculosis* and *M. orygis* (Fig. 3A). After performing a BLAST search of the amplicon, it was determined that this identical sequence is found in a variety of MTBC genomes (Fig. 3B). Taken together, we suggest that *M. orygis* was not previously identified in Pakistan because the majority of studies used the JB primers and reported positive samples as *M. bovis*. As a result, the prevalence and identity of the MTBC subspecies contributing to disease burden remained uncertain in Pakistan. The cause of bovine and zoonotic TB should be further investigated on a larger scale using diagnostic tools that are capable of reliably differentiating between MTBC subspecies.

To our knowledge, this study is the first of its kind reporting *M. orygis* from animals from Pakistan and the first study from Pakistan, which has deposited whole genome sequences from bTB cases. The findings of this study are concordant with previous reports that detected *M. orygis* in animals and humans within South Asia. Previously, *M. orygis* was reported in cattle and humans from South Asia (44) and in emigrants originating from South Asia (45–47). In Bangladesh, *M. orygis* was reported in cattle and monkeys, suggesting that *M. orygis* may have been introduced to Bangladesh with the import of cattle from Pakistan (12). Finally, a screening of 940 cultures in India and 715 publicly available genomes from South Asia identified several *M. orygis* isolates; there was a complete absence of *M. bovis* (28). Taken together, evidence from the literature along with the data from our study proves the endemicity of *M. orygis* across South Asia.

Another interesting finding from this study is the presence of *M. tuberculosis sensu stricto* in 8 (40%) of the 20 MTBC isolates identified in domestic bovines, highlighting the possibility for either reverse zoonosis or animal-to-animal transmission of *M. tuberculosis*. This requires further investigation as *M. tuberculosis* has not previously been reported as a prevalent cause of bTB in animals from Pakistan. However, *M. tuberculosis* has been reported in captive antelopes (48) and wild animals (49) in Pakistan. The isolation of *M. tuberculosis* from cattle has been reported in South India, and the cattle isolates were found to be phylogenetically related to isolates from farmers (50).

This study has several limitations. The first is that it relied on the presence of visible TB-like lesions or granulomas on the organ surfaces to identify samples for analysis. A postmortem examination is not a reliable method to identify all cases of bTB (51). Second, two isolates could only be identified as MTBC (Table 2) due to repeated failure of molecular assays to differentiate the samples. This was likely a result of low DNA concentrations and the quality of the samples. Another limitation was the disproportionate distribution of buffaloes and female animals relative to cattle and male animals. At the slaughterhouse selected for this study, the proportion of buffalo (89.25%) brought in for slaughtering was higher as compared to cattle (10.72%), resulting in the segregation of fewer tissues with TB-like lesions from cattle carcasses. The central region of the Punjab province has a higher buffalo population density due to different cultural preferences (52), which could result in the pooling of a higher number of buffalo in the slaughterhouse. Male animals are also usually slaughtered at a few months of age or raised as sacrificial animals for a religious occasion (Eid-Ul-Adha) (53). These factors might have caused an increased number of female animals in the slaughterhouse and, therefore, more females in our study. Finally, during this study, screening was performed on animals at a single slaughterhouse over a relatively short period of time, limiting the potential generalizability of the results to the rest of the country and highlighting a need for larger geographically representative surveys of bovine tuberculosis in the country.

We conclude that bTB was present in bovines in this slaughterhouse in Lahore and was caused by *M. orygis* and *M. tuberculosis sensu stricto*. Our results are in agreement with recent reports indicating that *M. orygis* is the predominant cause of bTB in South Asia. Detection of *M. tuberculosis* in bovines at our study site suggests that reverse zoonosis may have occurred, but transmission between animals is another possibility that warrants future exploration. A high proportion of *M. tuberculosis* in animals may have important implications in understanding the prevalence and risk of zoonotic TB in humans. Future studies identifying bovine or zoonotic TB in Pakistan should use molecular methods capable of reliably differentiating members of the MTBC. Collectively, our findings stress the importance of a One Health approach to manage tuberculosis in Pakistan.

## Data Availability

Sequences were deposited in the National Center for Biotechnology Information (NCBI) Sequence Read Archive (SRA) under BioProject accession number PRJNA958208.

## SUPPLEMENTAL MATERIAL

Supplementary Material 1 spectrum.02692-23-s0001.docx Supplemental materials and methods.

ASM does not own the copyrights to Supplemental Material that may be linked to, or accessed through, an article. The authors have granted ASM a non-exclusive, world-wide license to publish the Supplemental Material files. Please contact the corresponding author directly for reuse.
