# Supplementary material for: Molecular detection and characterization of the Mycobacterium tuberculosis complex subspecies responsible for bovine tuberculosis in Punjab, Pakistan
Source: Microbiol Spectr. 2024 Jan 16;12(2):e02692-23. doi: 10.1128/spectrum.02692-23 (PMC10846167; doi:10.1128/spectrum.02692-23)
Supplement: Supplementary Material 1 — Supplemental materials and methods. [file spectrum.02692-23-s0001.docx]

**Supplementary Methods:**

**Tissue samples containing TB-like lesions were triturated and DNA extraction was performed using the following protocol:**

Day 1:

1. Take a small quantity of tissue sample in pestle & mortar and add a small amount of DEPC treated water. Triturate properly until a fine mixture is obtained.
2. Transfer the mixture to a clean/autoclaved Eppendorf.
3. Centrifuge the mixture at 3000 rpm for 2 minutes.
4. Carefully transfer the supernatant to the new Eppendorf.
5. Centrifuge it at 14000 rpm for 14 minutes and then discard the supernatant.
6. Add 400ul Extraction buffer, 40 ul Proteinase k (20mg/ml), and 20 ul Lysozyme (10mg/ml) in Eppendorf containing the pellet.
7. Vortex mixture for a few seconds.
8. Incubated in the thermo-block at 56 C temperature overnight.

Day 2:

1. Add 500ul Phenol Chloroform Isoamyle Alcohol (PCI; 25:24:1) in Eppendorf and mix properly using a vortex.
2. Perform chilled Centrifugation at 14000 rpm for 15 minutes. Three layers will be created.
3. Carefully collect the uppermost layer into a separate Eppendorf.
4. Add 500ul chill Isopropyl alcohol, mix, and perform another chilled centrifugation at 14000 rpm for 10 minutes.
5. Discard the supernatant and add 1ml chilled absolute (>99.98%) Ethanol into the pellet.
6. Again centrifuged at 8000 rpm for 15 minutes.
7. Discard the supernatant and placed the Eppendorf in a thermo-block at 65 C until the pellet gets completely dried.
8. Re-suspended pellet in 20ul DNAse and RNAse-free water.
9. Gave heat shock at 60 C for 15-20 minutes.
10. Store extracted DNA at -20 C.

**PCR protocol for JB21 and JB22 primers:**

| Reaction volume  Initial denaturation  Denaturation for 35 cycles  Annealing  Elongation  Final elongation  Total number of cycles | 25μl  94°C for 5 minutes  94°C for 1 minute  55°C for 45 seconds  72°C for 1 minute  72°C for 10 minutes  35 |
| --- | --- |

**PCR protocol for RD9 and RD12 assays**

Master mix:

| 10X *Taq* buffer (Thermo Scientific)  Acetamide 50% (wt/vol)  MgCl_2_  dNTPs  *Taq* polymerase (Thermo Scientific)  Primers  Template DNA  Deionized water | 6.25μl  6.25μl  1.6mM  0.2mM  2.5 U  500nM  5μl  To 50μl |
| --- | --- |

Thermocycling conditions:

| Reaction volume  Initial denaturation  Denaturation for 35 cycles  Annealing  Elongation  Final elongation  Total number of cycles | 50μl  94°C for 3 minutes  94°C for 30 seconds  55°C for 1 minute  72°C for 1 minute  72°C for 10 minutes  35 |
| --- | --- |

**Supplementary Table 1** Primer sequences

| **Primers** | **Amplicon** |
| --- | --- |
| JB21 and JB221 primers  Forward: JB21 5-TCGTCCGCTGATGCAAGTGC´  Reverse: JB22 5-CGTCCGCTGACCTCAAGAAAG´ | 500bp |
| RD9 primers  Forward: CCGATACCATGCAACAACGG  Reverse 1: CGGTCTCTCCGAGCATTC  Reverse 2: GCTCGAGCTAGACCTGCAC | M. tb: 209bp  Non-M. tb MTBC: 410bp |
| RD12 primers  M. tb Forward 1: GTATTTGCGCCCATATCCTGG  M. tb Reverse 1: CCTGGCTTCAAGCACCATTC  M. bovis Forward 2: GGCCATCAACGTCAAGAACCTC  M. bovis Reverse 2: CGAACTCGTATTTTGTGGCCAC  M. orygis Forward 3: GTGGAAATGGAAGCGTTGACC  M. orygis Reverse 3: GGTACCTCCTCGATGAACCAC | M. tb: 409bp  M. bovis/BCG: 615  M. orygis: 264bp |
| Hsp65 primers  Forward: ACCAACGATGGTGTGTCCAT  Reverse: CTTGTCGAACCGCATACCCT | 500bp |
| 16S primers  Forward: AGAGTTTGATCCTGGCTCAG  Reverse: GTATTACCGCGGCTGCTG | 500bp |

**Supplementary Table 2** *M. orygis* sequences within the phylogenetic tree

| **Sample Name** | **Host** | **Country** |
| --- | --- | --- |
| ERR6375115 | Antelope | South Africa |
| ERR6375116 | Antelope | South Africa |
| ERR6375117 | Antelope | The Netherlands |
| ERR6375118 | Antelope | The Netherlands |
| ERR6375119 | Antelope | The Netherlands |
| ERR6375120 | Antelope | The Netherlands |
| ERR6375125 | Antelope | The Netherlands |
| ERR6375126 | Antelope | The Netherlands |
| SRR17254975 | Antelope | India |
| SRR17254976 | Antelope | India |
| SRR10251193 | Bison | India |
| SRR24255213 | Buffalo | Pakistan |
| SRR24255214 | Buffalo | Pakistan |
| SRR10251185 | Cattle | India |
| SRR10251186 | Cattle | India |
| SRR10251187 | Cattle | India |
| SRR10251188 | Cattle | India |
| SRR10251189 | Cattle | India |
| SRR10251190 | Cattle | India |
| SRR10251191 | Cattle | India |
| SRR10251192 | Cattle | India |
| SRR10251194 | Cattle | India |
| SRR10251197 | Cattle | India |
| SRR10251200 | Cattle | India |
| SRR10251201 | Cattle | India |
| SRR10251203 | Cattle | India |
| SRR17089002 | Cattle | India |
| SRR17089003 | Cattle | India |
| SRR20607504 | Cattle | India |
| SRR20622096 | Cattle | India |
| SRR20622097 | Cattle | India |
| SRR9157804 | Cattle | India |
| SRR10251195 | Deer | India |
| SRR10251198 | Deer | India |
| SRR10251199 | Deer | India |
| SRR10251202 | Deer | India |
| SRR17089001 | Deer | India |
| SRR17254977 | Deer | India |
| h37Rv__Lineage_4_ | Human | United Kingdom |
| ERR017782 | Human | Russia |
| ERR234675 | Human | Russia |
| ERR234676 | Human | Russia |
| ERR234682 | Human | Russia |
| ERR5336157 | Human | Norway |
| ERR5336158 | Human | Norway |
| ERR5336161 | Human | Norway |
| ERR5336162 | Human | Norway |
| ERR5336167 | Human | Norway |
| ERR6375123 | Human | Unknown |
| ERR6375124 | Human | Unknown |
| ERR6375127 | Human | Unknown |
| ERR6375128 | Human | Unknown |
| ERR6375129 | Human | Unknown |
| ERR6375130 | Human | Unknown |
| ERR6375131 | Human | Unknown |
| ERR6375132 | Human | Unknown |
| ERR6375133 | Human | Unknown |
| ERR6375134 | Human | Unknown |
| ERR6375135 | Human | Unknown |
| ERR6375136 | Human | Unknown |
| ERR6375139 | Human | Unknown |
| ERR6375140 | Human | Unknown |
| SRR16643349 | Human | Canada |
| SRR10321128 | Human | India |
| SRR10321130 | Human | India |
| SRR10321134 | Human | India |
| SRR10321138 | Human | India |
| SRR10321141 | Human | India |
| SRR10321143 | Human | India |
| SRR10321149 | Human | India |
| SRR10321152 | Human | India |
| SRR2100577 | Human | Unknown |
| SRR2101161 | Human | Unknown |
| SRR2101291 | Human | Unknown |
| SRR2101329 | Human | Unknown |
| SRR21691066 | Human | The Netherlands |
| SRR5642711 | Human | United States |
| SRR5642712A | Human | United States |
| SRR5642713A | Human | United States |
| SRR5642714 | Human | United States |
| SRR5642715 | Human | United States |
| SRR5642716 | Human | United States |
| SRR5642717A | Human | United States |
| SRR5642718 | Human | United States |
| ERR015582 | Unknown | Unknown |
| ERR2659153 | Unknown | Unknown |
| ERR2659154 | Unknown | Unknown |
| ERR2659155 | Unknown | Unknown |
| ERR2659156 | Unknown | Unknown |
| ERR6375121 | Unknown | Unknown |
| ERR6375122 | Unknown | Unknown |
| ERR6375137 | Unknown | Unknown |
| ERR6375138 | Unknown | Unknown |
| ERR6375141 | Unknown | Unknown |
| ERR6375142 | Unknown | Unknown |


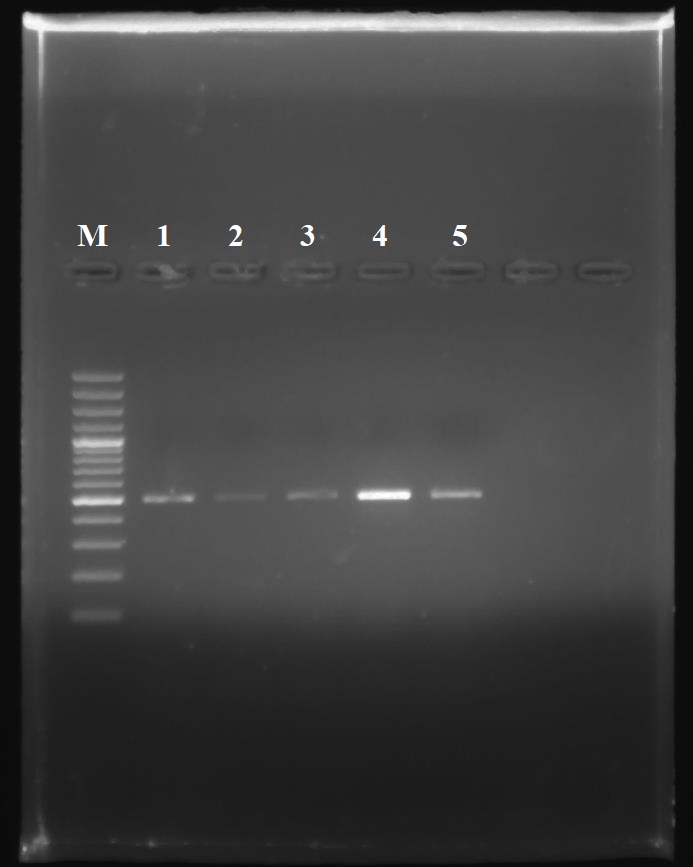


**Supplementary Figure 1** PCR screening targeting the 500bp fragment from the JB21 and JB22 primers. Lane M contains a 100bp ladder. Lanes 1-5: bR1, bR2, bR3, positive control, bR8.


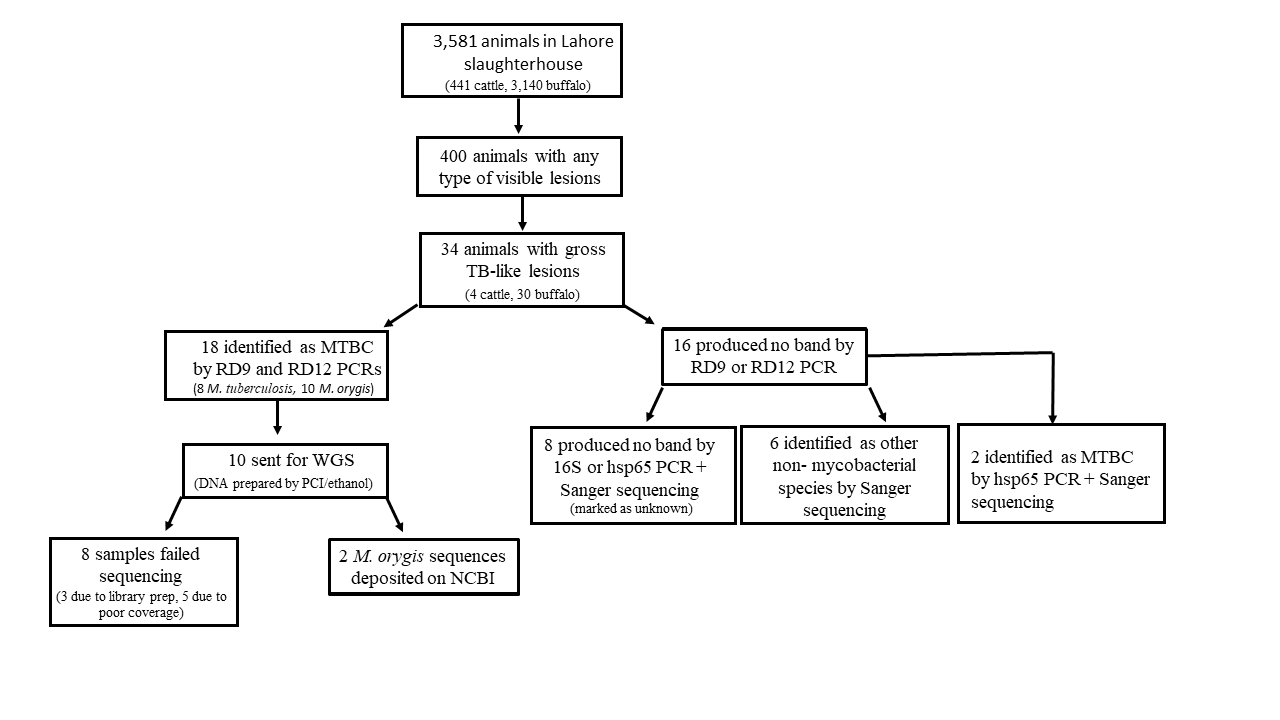


**Supplementary Figure 2** Sample flow chart.

**
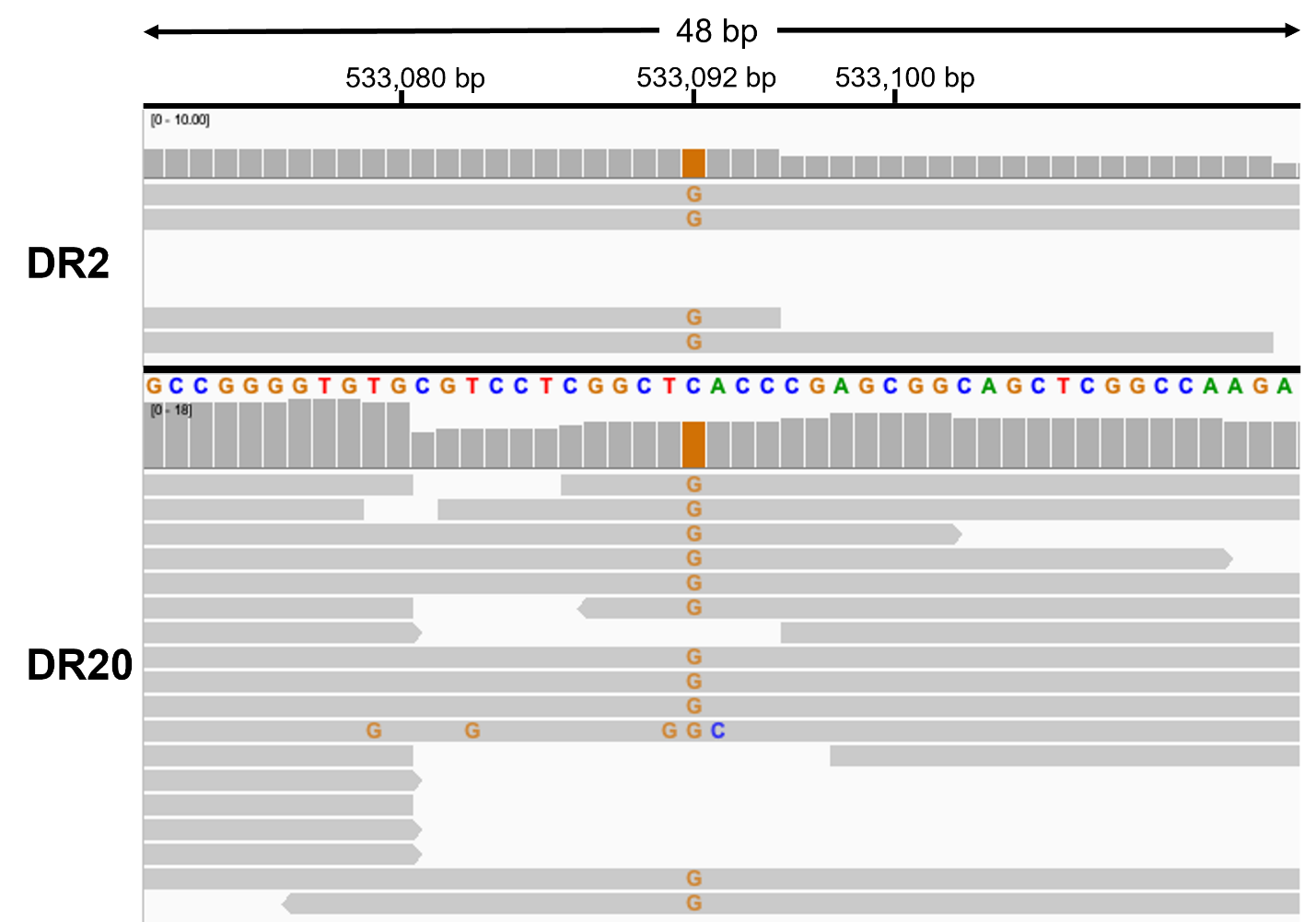
**

**Supplementary Figure 3** Visualization of *M. orygis*-specific SNP g698c on *Rv0444c*

Whole genome sequences from DR2 and DR20 were visualized on IGV. The g698c SNP on *Rv0444c* (nucleotide 533,092) was found at a 4X coverage in the DR2 sequence and a 12X coverage in the DR20 sequence


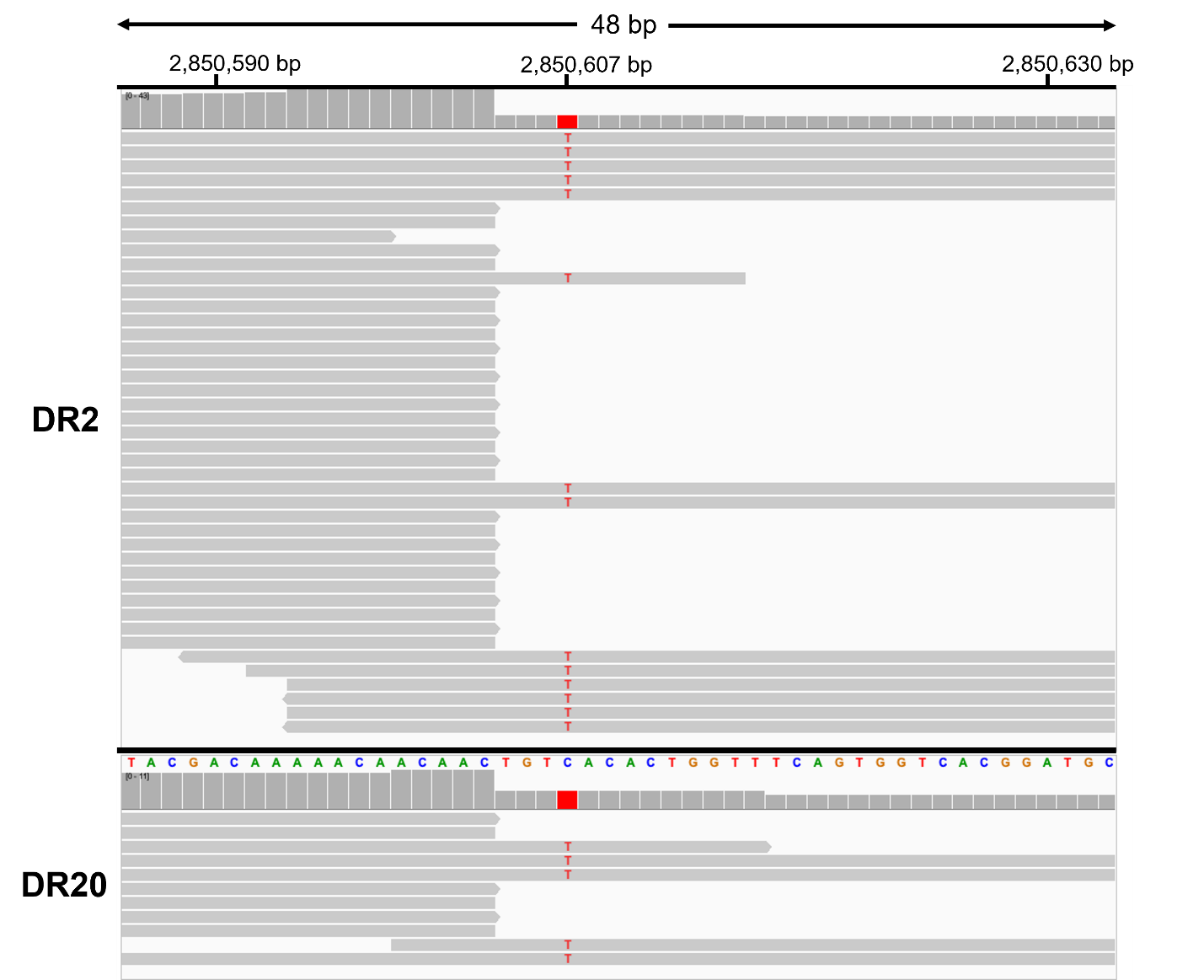


**Supplementary Figure 4** Visualization of *M. orygis*-specific C to T SNP at 2,850,067

Whole genome sequences from DR2 and DR20 were visualized on IGV. The C to T SNP located at nucleotide 2,850,067 was found at a 14X coverage in the DR2 sequence and a 5X coverage in the DR20 sequence.
